# Sternal Bone Marrow Harvesting and Culturing Techniques from Patients Undergoing Cardiac Surgery

**DOI:** 10.3390/mi12080897

**Published:** 2021-07-28

**Authors:** Jimmy J. H. Kang, Sabin J. Bozso, Ryaan EL-Andari, Michael C. Moon, Darren H. Freed, Jayan Nagendran, Jeevan Nagendran

**Affiliations:** 1 Division of Cardiac Surgery, University of Alberta, Edmonton, AB T6G 2B7, Canada; jkang@ualberta.ca (J.J.H.K.); bozso@ualberta.ca (S.J.B.); mmoon@ualberta.ca (M.C.M.); dhfreed@ualberta.ca (D.H.F.); jayan@ualberta.ca (J.N.); 2 Faculty of Medicine and Dentistry, University of Alberta, Edmonton, AB T6G 2B7, Canada; elandari@ualberta.ca

**Keywords:** bone marrow harvest, mesenchymal stromal cells, isolation and culturing, tissue engineering, stem

## Abstract

Background: Mesenchymal stromal cells (MSCs) are the most prominent cell type used in clinical regenerative medicine and stem cell research. MSCs are commonly harvested from bone marrow that has been aspirated from patients’ iliac crest. However, the ethical challenges of finding consenting patients and obtaining fresh autologous cells via invasive extraction methods remain to be barriers to MSC research. Methods: Techniques of harvesting sternal bone marrow, isolating and culturing MSCs, MSC surface phenotyping, and MSC differentiation are described. Samples from 50 patients undergoing a sternotomy were collected, and the time taken to reach 80% confluency and cell count at the second splitting of MSC were measured. Results: MSC isolated from the sternal bone marrow of patients undergoing cardiac surgery demonstrated successful MSC surface phenotyping and MSC differentiation. The mean cell count at the time of the second split was 1,628,025, and the mean time taken to reach the second split was 24.8 days. Conclusion: Herein, we describe the first reported technique of harvesting sternal bone marrow from patients already undergoing open-chest cardiac surgery to reduce the invasiveness of bone marrow harvesting, as well as the methods of isolating, culturing, and identifying MSCs for the clinical application of constructing autologous MSC-derived biomaterials.

## 1. Introduction

Mesenchymal stromal cells (MSCs) (also known as mesenchymal stem cells, or multipotent mesenchymal stromal cells [1]) are the most prominent cell type used in clinical regenerative medicine and stem cell research today. Over 200 active MSC clinical trials [2] have been performed and have continued to rise exponentially since 2004 [3]. Despite being in the early stages of development with limited clinical applications, MSC’s descriptions of its multipotency, immunomodulatory, and trophic effects [4,5] have garnered interest among researchers and clinicians to explore MSC therapy in treating a wide variety of conditions. These conditions include immune-mediated diseases such as the graft versus host disease [6] and regenerating damaged tissues of mesodermal origin such as the heart [7,8], bones [9], and cartilages [10,11], as well as the tissue engineering of patient-specific biomaterials [2,12,13].

MSCs were originally described by Freidenstein et al. in the early 1970s through a series of papers describing the isolation of clonal fibroblastic cells from bone marrow, using its ability to adhere to plastic culture vessels [14,15,16]. Their capability to differentiate into osteogenic cells was later developed by Pittinger et al. in 1999, where they successfully differentiated the colony of cells into adipocytic, chondrocytic, and osteocytic lineages [17]. Since then, scientists have been able to successfully isolate MSC from bone marrow [18], dental pulp [19], adipose tissue [20], and umbilical cords [21], and protocols have also been developed for proper cultivation and multilineage differentiation [21,22,23]. In 2006, the International Society of Cellular Therapy (ISCT) further established three minimal criteria for defining MSCs [1]. First, MSCs must be plastic adherent under standard culturing conditions. Second, they must be positive for CD105, CD73, and CD90, and negative for CD45, CD34, CD14 or CD11b, CD79alpha or CD19, and HLA-DR surface molecules. Lastly, they must differentiate into adipocytes, osteoblasts, and chondroblasts in vitro.

Despite the structured guidelines in place, the ethical challenges of finding consenting patients and obtaining fresh autologous cells via invasive extraction methods still remain to be barriers of MSC research. Recent developments in perivascular MSC extraction using adipose tissues has attempted to remedy this issue, but the invasive nature of the procurement of bone marrow presents a significant challenge for human MSC study [24]. Herein, we describe the first reported method of harvesting bone marrow from the sternum of patients who are undergoing open-chest cardiac surgery. Ironically, this is the least invasive method of collecting bone marrow for research, as the sample collection is done from patients already scheduled to undergo sternotomy. The only other reported methods of obtaining autologous bone marrow are from iliac crest aspirations [25], sternum aspirations [26], and femur rasping during hip replacements [27]. Furthermore, our protocol and results on MSC culturing, phenotyping, and differentiation using sternal bone marrow will be described in this review.

## 2. Materials and Methods

### 2.1. Initial Sample Collection

**Figure 1:**
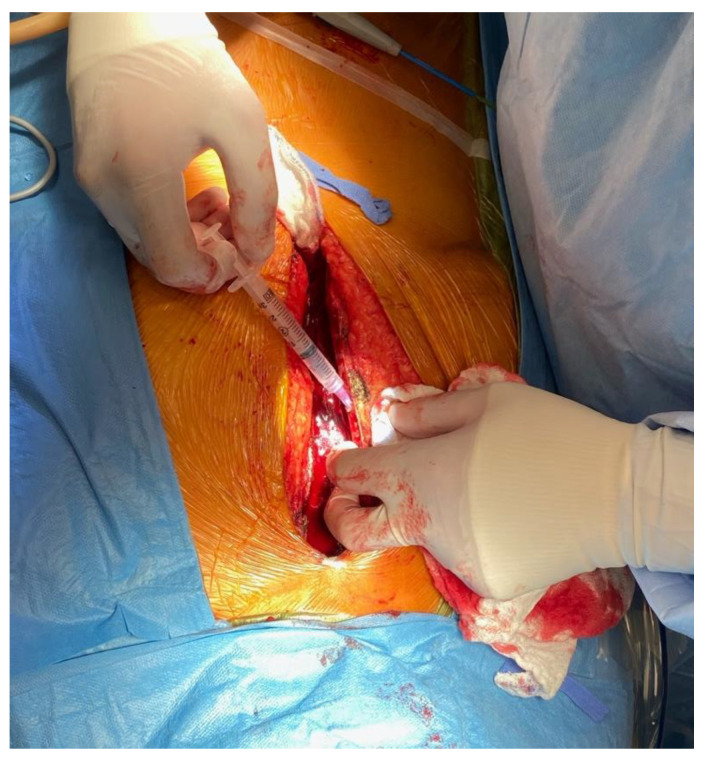
Sternal bone marrow harvest with a 5 mL blunt-tip syringe following a sternotomy.

Fifty patients aged 50–90 years who were undergoing cardiac surgery and a planned sternotomy or hemi-sternotomy consented to bone marrow collection. Patients who had active hematological malignancy or were receiving corticosteroids were excluded from the study. Written informed consent forms for participation in the research containing the purpose of the study, procedural details, risks and benefits, and confidentiality were carefully explained to the patients prior to surgery. Human study was approved by the Health Research Ethics Board and ARISE (Alberta Research Information Services). Once the sternotomy was performed and hemostasis was achieved with cautery to the edges of the sternum, a 5 mL syringe with a blunt needle tip or a surgical curettage were used to collect the discharging bone marrow from between the edges just below the manubrium (Figure 1). A volume of 0.3–1 mL of bone marrow was collected. Once drawn, the sample was stored at room temperature not exceeding 4 h.

### 2.2. MSC Isolation and Culturing

A complete Dulbecco’s Modified Eagle Medium Nutrient Mixture (DMEM F12) supplemented with 20% Fetal Bovine Serum (FBS), 100 mM ascorbic acid, and 100 μg/mL Primocin was prepared. The completed media was warmed in a 37 °C water bath. The 5 mL syringe, the complete DMEM F12 container, and a 75 cm^2^ cell culture flask with a vented cap was sprayed with 70% ethanol before placing them in a laminar flow hood. Using an electronic pipette controller, 15 mL of the complete DMEM F12 was pipetted into the culturing flask. The bone marrow sample from the syringe was carefully pushed into the flask, being sure to draw any large clumps of marrow and residual bone back into the syringe and then push it out again to spread the sample around the flask. The flask was gently shaken in an up and down and sideway motion to ensure the sample had covered the bottom of the flask. The flask was placed in a 37 °C, 5% CO_2_ incubator for >18 h. The media was aspirated at the corner of the flask with a vacuum pipette to suction off any media and samples that had not adhered to the plastic. The adhered cells on the flask were washed with 15 mL of 1X PBS. Then, 1X PBS was aspirated at the corner of the flask. A fresh 15 mL of the complete DMEM F12 was added into the culturing flask and incubated further for >18 h. Confluency of the MSCs was checked under a light microscope. Washing with PBS and incubation with media was done every 3 days until the confluency had reached 85%.

### 2.3. MSC Expansion

Old DMEM F12 media were aspirated and the cells were washed with 15 mL of 1X PBS. A volume of 15 mL of 10X TrypLE solution that had been sitting in a 37 °C water bath for 20 min was added to the flask to lift the plastic adhered MSCs. The flask was incubated in a 37 °C, 5% CO_2_ incubator for 30 min. MSCs were checked to see if they had lifted off of the flask using a light microscope. A volume of 15 mL of complete DMEM F12 that had been sitting in a 37 °C water bath was added to the flask. The contents in the flask were pipetted into a 50 mL conical tube and centrifuged at 400× *g* for 15 min at 18 °C. The supernatant was removed without disturbing the pellet. The pellet was resuspended with 20 mL of 1X PBS and mixed to disrupt the pellet, and then centrifuged under the same setting. The supernatant was removed, and the cells were resuspended with complete DMEM F12 of appropriate volume and the cells were seeded into the desired number of flasks containing 15 mL of complete DMEM F12. The centrifugation and resuspension were repeated until the second iteration of MSC expansion.

### 2.4. Surface Phenotyping

MSCs were detached from the plastic using TrypLETM Select 10× and resuspended at a concentration of 1 × 10^7^ cells/mL in BD Pharmingen™ Stain Buffer. They were added to labelled tubes with the antibodies from the BD Stemflow™ Human MSC Analysis Kit: FITC Mouse Anti-Human CD90 (5 µL); PE Mouse Anti-Human CD44 (5 µL); PerCP-Cy™ 5.5 Mouse Anti-Human CD105 (5 µL); APC Mouse Anti-Human CD73 (5 µL); hMSC Positive Isotype Control Cocktail (20 µL); PE hMSC Negative Isotype Control Cocktail (20 µL); hMSC Positive Cocktail (20 µL) containing CD90 FITC, CD105 PerCP-Cy™ 5.5, CD73, and APC; and PE hMSC Negative Cocktail (20 µL) containing CD34 PE, CD11b PE, CD19 PE, CD45 PE, and HLA-DR PE. Volumes of 100 µL of the prepared cell suspension were added to the tubes outlined in step 2. The tubes were incubated in the dark for 30 min on ice. The cells were washed twice with BD Pharmingen™ Stain Buffer and resuspended at 300–500 µL in BD Pharmingen™ Stain Buffer and analyzed on a flow cytometer.

### 2.5. Adipogenesis Differentiation

A complete Adipogenesis Differentiation Medium comprised of 90 mL StemPro^®^ Adipocyte Differentiation Basal Medium 1× concentration, 10 mL StemPro^®^ Adipocyte Supplement 1× concentration, and 50 µL Gentamicin reagent 5 µg/mL was prepared. MSCs from passage 1 were collected and resuspended with the appropriate amount of pre-warmed complete DMEM F12 medium from Basic Protocol 1. MSCs were seeded onto a 12-well plate at 1 × 10^4^ cells/cm^2^. After 1–4 days of incubation at 37 °C, 5% CO_2_, complete DMEM F12 media was replaced with Complete Adipogenesis Differentiation Medium and the incubation continued for 7–14 days with refeeding every 3 days. After 7–14 days of incubation, the cells were fixed with 4% PFA solution for 30 min. The cultures were rinsed with PBS x1 solution twice and LipidTOXTM was added to the cultures to stain the lipid vacuoles for 15–30 min. DAPI stain was then added for counterstain. Images were captured under a fluorescent microscope for qualitative analysis.

### 2.6. Osteogenesis Differentiation

A complete Osteogenesis Differentiation Medium comprised of 90 mL StemPro^®^ Osteocyte/Chondrocyte Differentiation Basal Medium 1× concentration, 10 mL StemPro^®^ Osteogenesis Supplement 1× concentration, and 50 µL Gentamicin reagent 5 µg/mL was prepared. MSCs were collected from passage 1 and resuspended with the appropriate amount of pre-warmed complete DMEM F12 medium from Basic Protocol 1. MSCs were seeded onto a 12-well plate at 5 × 10^3^ cells/cm^2^. After 1–4 days of incubation at 37 °C, 5% CO_2_, growth media was replaced with Complete Osteogenesis Differentiation Medium and the incubation continued for >21 days with refeeding every 3 days. After >21 days of incubation, the cells were fixed with 4% PFA solution for 30 min and then rinsed twice with distilled water, and then 2% Alizarin Red S staining solution was added for 2–3 min. Images were captured under a light microscope for qualitative analysis.

### 2.7. Chondrogenesis Differentiation

A complete Chondrogenesis Differentiation Medium comprised of 90 mL StemPro^®^ Osteocyte/Chondrocyte Differentiation Basal Medium 1× concentration, 10 mL StemPro^®^ Chondrogenesis Supplement 1× concentration, and 50 µL Gentamicin reagent 5 µg/mL was prepared. MSCs from passage 1 were collected and resuspended with the appropriate amount of pre-warmed complete DMEM F12 medium from Basic Protocol 1. Micro mass cultures of 1.6 × 10^7^ cells/mL were generated by seeding 5 μL droplets of cell solution on a 12-well plate. Complete chondrogenesis media was added to the wells after 2 h under high humidity conditions and refed every 3 days. After 14 days of incubation at 37 °C, 5% CO_2_, the cells were rinsed with PBS and were fixed with 4% PFA solution for 30 min. Next, 1% Alcian Blue staining solution was added to 0.1 N HCL for 30 min and the cells were visualized under a light microscope for qualitative analysis.

### 2.8. MSC Expansion Timing and Cell Count

MSCs were isolated and expanded from the sternal bone marrow of 50 patients undergoing a sternotomy at the Mazankowski Heart Institute, Edmonton, Alberta, Canada. Patients were divided into 10-year age groups, and the number of days taken for MSC to reach 80% confluency was recorded. The MSC were counted after using 10X Trypsin to lift the cells from the plastic. A cell counter was used to visualize the cells under a light microscope. The full list of media and solutions used in our protocols can be found in Supplementary Table S1.

## 3. Results

### 3.1. Surface Phenotyping

**Figure 2:**
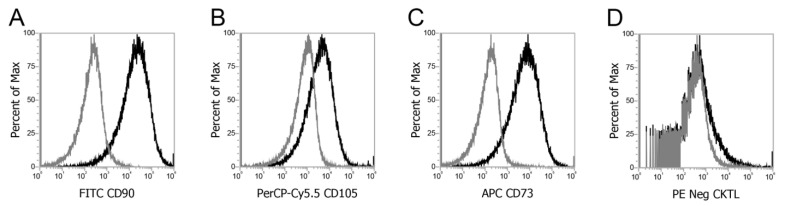
Surface phenotyping under flow cytometry for positive cell surface antigen expression for CD 90 (**A**), CD105 (**B**), and CD 73 (**C**) and absence of negative cell surface antigens in a negative cocktail of CD 34, CD 11b, CD19, and CD45, and HLA-DR (**D**) expression in bone marrow-derived MSCs.

Figure 2 demonstrates successful surface phenotyping of MSC under flow cytometry for proliferation of positive cell surface antigens CD 90 (A), CD105 (B), and CD 73 (C) and the absence of negative cell surface antigens in a negative cocktail of CD 34, CD 11b, CD19, and CD45, and HLA-DR (D) expression in bone marrow-derived MSCs.

### 3.2. MSC Differentiation

**Figure 3:**
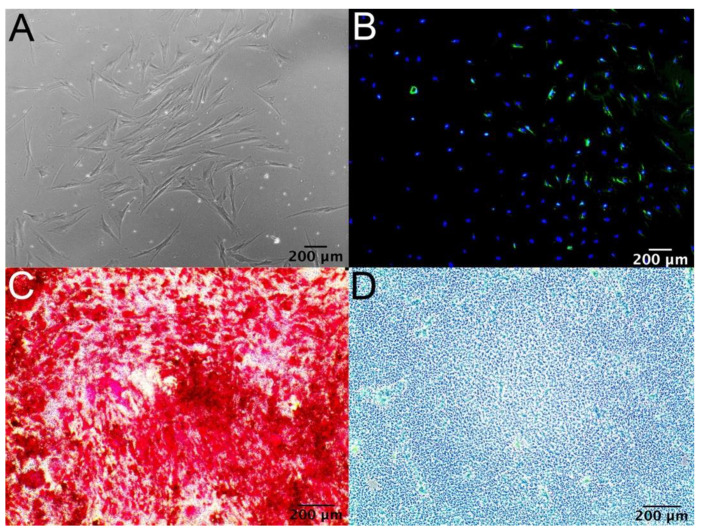
(**A**) Micrograph representing bone marrow-derived MSCs. (**B**) Adipocyte differentiation of MSCs with LipidTOXTM Stain. (**C**) Osteoblast differentiation of MSCs with Alizarin Red S stain. (**D**) Chondrocyte differentiation of MSCs with Alcian Blue stain.

Figure 3 depicts successful differentiation of MSC (Figure 3A) into adipocytes under LipidTOXTM Stain (Figure 3B), osteoblasts under Alizarin Red S stain (Figure 3C), and chondrocytes under Alcian Blue stain (Figure 3D) using the differentiation protocol described in the methods and materials section.

### 3.3. MSC Expansion Timing and Cell Count

**Figure 4:**
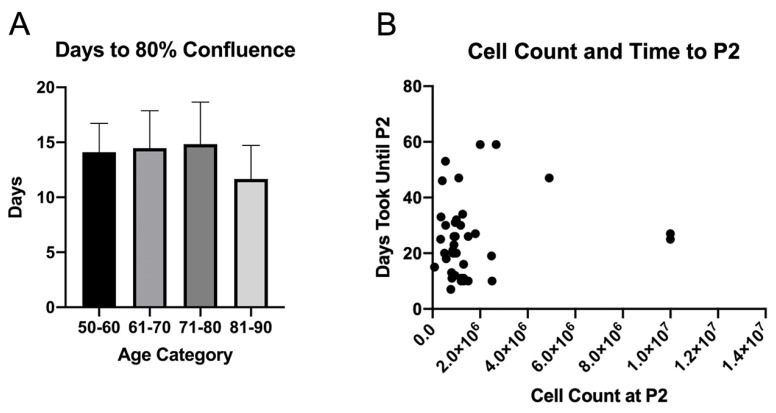
(**A**) Time taken for MSCs to reach 80% confluency since the date of collection stratified based on age groups (50–60, n = 9; 61–70, n = 19; 71–80, n = 19; 80–90, n = 3). (**B**) Cell count at the time of P2 and amount of time until P2 split of MSCs.

MSC were isolated and expanded from the sternal bone marrow of 50 patients. The patients were divided into different age groups (50–60, n = 9; 61–70, n = 19; 71–80, n = 19; 80–90, n = 3). There was no statistically significant difference in the number of days until the first split of MSC cells at 80% confluency between the different patient age groups (p = 0.523). The 50–60 age group reached 80% confluency in 14.1 ± 2.6 days, 61–70 in 14.5 ± 3.4 days, 71–80 in 14.8 ± 3.8 days, and 81–90 in 11.7 ± 3.1 days (Figure 4A). For 40 of our samples, the mean MSC count at the time of the second split at 80% confluency was 1,628,025 and the mean time taken to reach P2 was 24.8 days. (Supplementary Table S2). The first 10 samples collected were used for other experiments in the lab and have been excluded from Figure 4B.

## 4. Discussion

Autologous bone marrow harvest for non-clinical research has been strictly limited due to the significant ethical challenge associated with the invasive nature of the procedure. Herein, we demonstrate the first reported case of using sternal bone marrow from patients already undergoing a sternotomy for cardiac surgery. The collection of bone marrow using a surgical curettage or a blunt-tip syringe from an open sternum bypasses the ethical challenge of causing additional risk to the patient, as they are already scheduled to undergo cardiac surgery. Biopsy or collection of bone marrow from sternums via aspirations in humans have been described previously as an alternative to iliac crest aspirations [26,28,29]; however, the possibility of serious complications such an aortic dissection [30] and pericardial tamponade [31] have limited this practice to experienced clinicians. It was also reported to induce significant anxiety and pain among patients [32]. In contrast, bone marrow collection from an open sternum during cardiac surgery is a controlled procedure that does not pose any additional risks to the patient. This study also highlights a new set of patient population that MSC could be harvested from for both autologous and allogenic MSC studies.

While immediate therapeutic application of autologous MSCs from the time of collection may be limited due to the quantity of the sample collected, adequate culturing and expansion of MSCs following the harvest can still be achieved in a timely manner outlined in Figure 4. First, MSC samples from 40 patients took 24.8 days to reach a mean cell count of 1,628,025. Second, there was no significant difference in the number of days taken to reach 80% confluency across all age groups, which demonstrates clinical applicability in using autologous samples from the elderly to use MSC for research purposes such as building MSC-derived biomaterials. Our results with 0.3–1.0 mL of initial bone marrow collected generated a lower number of MSC when compared groups that use large amounts of iliac crest bone marrow aspirate, but we demonstrate that over a million MSCs can be generated with fraction of bone marrow collected in a similar time frame. Lubis et al. showed that with 30 mL of bone marrow aspirate from the iliac crest of nine patients, the time taken to reach a mean of 12.14 × 10^6^ cells was 28 days [33]. In addition, with the collected marrow and isolated MSC, we were still able to meet the ISCT guidelines for MSC definition, which require 1 × 10^7^ MSC cells for surface phenotyping (Figure 2) and MSC differentiation into adipocytic, osteocytic, and chondrocytic lineages (Figure 3).

Sternal bone marrow harvest and various applications of autologous MSCs currently have basic and translational research applications specifically in tissue engineering small biomaterials. We have previously shown that bovine pericardial tissue-engineered heart valves that are reseeded with autologous MSCs have a reduced xenoreactive immune response compared to native bovine pericardial valves when exposed to autologous human blood [34]. We first decellularized the native bovine pericardial tissue using detergents to leave only the extracellular matrix scaffold, and then recellularized the valve with human MSCs which we cultured from the sternal bone marrow. Successful tissue engineering of bioprosthetic valves that are not immunogenic would mean that a large population of patients currently receiving xenogenic bovine bioprosthetic aortic valves may not require re-operation, as tissue-engineered valves could protect against xenogenic reactions that induce the failure of bioprosthetic valves. This study also shows the feasibility of conducting basic science and translational research using a minute quantity of bone marrow obtained from the sternum in addition to the already reported method of extracting and processing bone marrow aspirations in orthopedic settings using posterior superior iliac spine with 30–120 mL of bone marrow aspirate [25,35,36,37].

The future directions from the application of our protocol are not limited to tissue engineering. Elgaz et al. and Kim et al. have shown that MSCs have immune modulatory and anti-inflammatory properties which allow for a reduction in immune response to a variety of implanted grafts [38,39]. The incorporation of MSCs with various tissues or devices may attenuate immune responses and increase the durability of implanted materials. Singh et al. and Golpaninan et al. have also demonstrated remodelling of multiple systems including the myocardium, allowing for regeneration of damaged or fibrosed myocardial tissue [40,41]. Kim et al. and Castro-Manrreza el al. have also demonstrated the utility of MSCs in autoimmune diseases, hepatic dysfunction, and malignancy among others [39,42]. While the potential utility of MSCs is vast, it is important to identify efficient and effective ways to isolate and culture these cells. Our described method allows for the collection and culture of MSCs without an additional invasive procedure and using a relatively small amount of collected marrow. Furthermore, while the patient population who may clinically benefit from our MSC harvesting technique may be currently limited to those getting a sternotomy, this protocol describes an entirely new set of patient population from which MSCs could be harvested from for basic and translational research for both autologous and allogenic studies.

Limitations from this study include collection of minute quantities of sternal bone marrow, which made the quantification of the initial sample unavailable (0.3–1.0 mL). The hemostasis of the sternal bone marrow is conventionally achieved immediately following a sternotomy with bone wax at our institution. Despite patient consent, the patient’s surgery is the priority and the collection of bone marrow had to be achieved fairly quickly before the application of the bone wax. Despite the curettage and suction with a blunt-tip syringe, bone marrow collection was limited to 0.3–1.0 mL per patient. Due to the bone marrow containing fragments of bone scraped from the sternum and collected with the blunt-tip syringe, the initial sample collected could not be measured accurately and quantitatively assessed. Thus, the contents were immediately transferred over to the lab and plated in the 75 cm^2^ cell culture flask without going through an additional step of cell quantification. This would explain some of the variability seen in the cell count and the amount of time until the first split at 80% confluency. We remedied this limitation by having a relatively large sample size of patients compared to other studies of the same topic in the literature. In addition, we propose that the differences in the MSC cell count that were seen in our minute quantities of bone marrow obtained are of lesser significance when comparing our study to iliac crest bone marrow studies where volumes of 30 mL of marrow were sampled. Our culturing technique may also differ from other labs using a different formation of cell culture media. Although several studies have shown the usage of 20% FBS on human MSC culturing [43,44,45,46], others have shown that 10% FBS also works effectively on culturing [47,48,49,50]. The usage of human serum versus FBS remains contentious, as studies have heterogenous bone marrow sample harvest sites, donor sera, and sample numbers [45]. When comparing the proliferative effects of human mesenchymal cells, Kuznetsov et al. [46] showed superiority of FBS over human serum [46], while Yamamoto et al. [50] and Spees et al. [44] showed similar effects. On the other hand, Stute et al. [49], Shahdadfar et al. [48] and Kobayashi et al. [47] have demonstrated increased proliferation of human MSC with human serum use over FBS. With an increased concentration of FBS and primocin, there is a risk of mycoplasma contamination, thus the proliferation of MSC should always be weighed against the risk of contamination. In addition strict rules for a good aseptic technique must be followed, and the sources of aerosol contaminations should be carefully controlled.

## 5. Conclusions

We have demonstrated the first reported method of bone marrow harvest from the sternums of patients undergoing cardiac surgery. Bone marrow collection was mainly limited to aspirations of the iliac crest and sternum. With the ethical challenges of causing undue harm to patients, the usage of autologous MSCs for research purposes has been limited. We demonstrate that even with a small quantity of autologous bone marrow collected and regardless of patient age, construction and testing of MSC-derived biomaterials such as tissue-engineered heart valves is possible for future clinical applications.

## Supplementary Materials

The following are available online at https://www.mdpi.com/article/10.3390/mi12080897/s1, Table S1: Media and solutions used in our protocols, Table S2: Cell Count and Time Took to P2 Split of MSCs since Harvest.
