# Supplementary material for: Sternal Bone Marrow Harvesting and Culturing Techniques from Patients Undergoing Cardiac Surgery
Source: Micromachines (Basel). 2021 Jul 28;12(8):897. doi: 10.3390/mi12080897 (PMC8397946; doi:10.3390/mi12080897)
Supplement: Supplementary file 1 [file micromachines-12-00897-s001.zip › micromachines-1304283-supplementary.pdf]

Table S1. Media and solutions used in our protocols.

Ingredient	Company, Cat. No.	Final Conc.	Comment
**Alcian-Blue Staining Solution**			
Alcian-Blue[a]	Millipore Sigma, TMS-010-C	1%	-
**Adipogenesis Differentiation Medium**			
StemPro® Adipogenesis Differentiation Basal Medium[b]	ThermoFisher, A10410-01	1X	-
StemPro® Adipogenesis Supplement[c]	ThermoFisher, A10065-01	1X	-
Gentamicin Reagent	ThermoFisher, 15710	5 µg/mL	-
**Alizarin Red S Staining Solution**			
Alizarin Red S[d]	Millipore Sigma, A5533-25G	2%	-
**Chondrogenesis Differentiation Medium**			
StemPro® Osteocyte/Chondrocyte Differentiation Basal Medium[b]	ThermoFisher, A10069-01	1X	-
StemPro® Chondrogenesis Supplement[c]	ThermoFisher, A10064-01	1X	-
Gentamicin Reagent	ThermoFisher, 15710	5 µg/mL	-
**Complete Medium**[e]			
Dulbecco's Modified Eagle Medium Nutrient Mixture DMEM F12	ThermoFisher, 11330-032	-	-
Fetal Bovine Serum, Qualified Standard[f]	ThermoFisher, 12483-020	20%	-
Ascorbic Acid, Reagent Grade, LabChem™[d]	Fisher Scientific, LC115309	100 mM	-
Primocin™[g]	InvivoGen, ant-pm-1	100µg/mL	-
**LipidTox Stain**			-
HCS LipidTOX™ Green Neutral Lipid Stain[h]	InvivoGen, H34475	1:100 dilution	Prepare a volume to completely cover cells
**Osteogenesis Differentiation Medium**			-
StemPro® Osteocyte/Chondrocyte Differentiation Basal Medium[b]	ThermoFisher, A10069-01	1X	-
StemPro® Osteogenesis Supplement[c]	ThermoFisher, A10066-01	1X	-
Gentamicin Reagent	ThermoFisher, 15710	5 µg/mL	-
**Paraformaldehyde (PFA)** 4%[i]			-
16% Formaldehyde	ThermoFisher, 28906	4%	Dilute in PBS ThermoFisher, 70011044
**Staining Buffer Solution**[j]			-
Stain Buffer (FBS)	BD Pharmingen™, 554656	-	-
**Trypsin Solution**			-
TrypLE™ Select 10x[k]	ThermoFisher, A12177-02	-	-

[a] Store at room temperature up to 4 months from date of receipt.
[b] Store at 2°C to 8°C; protected from light for up to 12 months.
[c] Store at -20°C to -5°C in the dark for up to 12 months.
[d] Store at room temperature
[e] Store at 4°C and warm in 37°C water bath for 15-20 min before use.
[f] Store at -20°C and warm in 37°C water bath for 15-20 min before use.
[g] Store at 4°C for 3 months or at -20 °C for long-term storage
[h] Store at -20°C protected from light
[i] Store at -20°C in 1ml aliquots and use within 6 months
[j] Store undiluted at 4°C
[k] Store at 15°C to 30°C protected from light for up to 24 months

Table S2. Cell Count and Time Took to P2 Split of MSCs since Harvest

Patient Number	Cell Count at P2	Days Took to P2
Patient 1	356,000	33
Patient 2	410,000	46
Patient 3	800,000	13
Patient 4	940,000	12
Patient 5	337,000	25
Patient 6	1,300,000	16
Patient 7	900,000	23
Patient 8	550,000	30
Patient 9	1,300,000	10
Patient 10	1,200,000	10
Patient 11	2,500,000	10
Patient 12	1,000,000	20
Patient 13	570,000	18
Patient 14	1,300,000	11
Patient 15	500,000	20
Patient 16	1,200,000	11
Patient 17	10,000,000	27
Patient 18	10,000,000	25
Patient 19	812,000	11
Patient 20	1,500,000	10
Patient 21	768,000	7
Patient 22	1,500,000	26
Patient 23	1,260,000	34
Patient 24	2,480,000	19
Patient 25	1,000,000	32
Patient 26	1,800,000	27
Patient 27	905,000	20
Patient 28	80,000	15
Patient 29	940,000	12
Patient 30	955,000	26
Patient 31	860,000	20
Patient 32	953,000	31
Patient 33	900,000	26
Patient 34	850,000	21
Patient 35	1,180,000	30
Patient 36	2,000,000	59
Patient 37	2,675,000	59
Patient 38	540,000	53
Patient 39	1,100,000	47
Patient 40	4,900,000	47
Mean	1,628,025	25
Median	977,500	22
